# Representational similarity analysis reveals task-dependent semantic influence of the visual word form area

**DOI:** 10.1038/s41598-018-21062-0

**Published:** 2018-02-14

**Authors:** Xiaosha Wang, Yangwen Xu, Yuwei Wang, Yi Zeng, Jiacai Zhang, Zhenhua Ling, Yanchao Bi

**Affiliations:** 1 College of Information Science and Technology, Beijing Normal University, Beijing, 100875 China; 2 National Key Laboratory of Cognitive Neuroscience and Learning & IDG/McGovern Institute for Brain Research, Beijing Normal University, Beijing, 100875 China; 3 Beijing Key Laboratory of Brain Imaging and Connectomics, Beijing Normal University, Beijing, 100875 China; 4 Research Center for Brain-inspired Intelligence & National Laboratory of Pattern Recognition, Institute of Automation, Chinese Academy of Sciences, Beijing, 100190 China; 5 Center for Excellence in Brain Science and Intelligence Technology, Chinese Academy of Sciences, Shanghai, 200031 China; 6 National Engineering Laboratory for Speech and Language Information Processing, University of Science and Technology of China, Hefei, 230027 China

## Abstract

Access to semantic information of visual word forms is a key component of reading comprehension. In this study, we examined the involvement of the visual word form area (VWFA) in this process by investigating whether and how the activity patterns of the VWFA are influenced by semantic information during semantic tasks. We asked participants to perform two semantic tasks - taxonomic or thematic categorization - on visual words while obtaining the blood-oxygen-level-dependent (BOLD) fMRI responses to each word. Representational similarity analysis with four types of semantic relations (taxonomic, thematic, subjective semantic rating and word2vec) revealed that neural activity patterns of the VWFA were associated with taxonomic information only in the taxonomic task, with thematic information only in the thematic task and with the composite semantic information measured by word2vec in both semantic tasks. Furthermore, the semantic information in the VWFA cannot be explained by confounding factors including orthographic, low-level visual and phonological information. These findings provide positive evidence for the presence of both orthographic and task-relevant semantic information in the VWFA and have significant implications for the neurobiological basis of reading.

## Introduction

The left posterior occipitotemporal sulcus is a key region in the neural circuitry of reading. It is consistently activated by visual words across various writing systems^1,2^, adapts to repeated presentation of words^3–5^ and captures orthographic similarity among words^6–8^. Its sensitivity to visual words develops with reading acquisition^9,10^ and decreases upon damage^11,12^. All these lines of evidence indicate the involvement of this region in orthographic representation and justify its name as the “visual word form area” (VWFA)^13^.

A central goal of reading is mapping word forms onto word meanings for comprehension^14^. Because of its structural and functional connections with higher-order language regions^9,15,16^, the VWFA is commonly assumed to play an essential role in such mapping^17,18^. The exact mechanism of the form-meaning mapping and whether the VWFA activity is modulated by word semantic properties remain inconclusive. Significant semantic priming effects were found in this region in a visual lexical decision task^19^, but not in a similar paradigm with a semantic oddball detection task^3^ or a naming task^4^. The effects that semantically related word pairs showed more similar activity patterns than semantically unrelated ones in the VWFA were found to be marginally significant in one recent study^7^, but not in an earlier study^20^.

One possible explanation for these inconsistent findings of semantic effects in the VWFA might be related to the multidimensional organization of the semantic space^21–23^. Previous studies examined semantic effects by contrasting conditions of strong and weak semantical relatedness, with relatedness quantified from subjective association strength^3^ or computational linguistics^7^. Nevertheless, concepts can be related to each other in very different ways. For example, taxonomic and thematic relations are two dissociable types of relations in the semantic system^24^, with the former based on shared features (e.g. teacher-doctor) and the latter on frequent co-occurrence in events (e.g. teacher-classroom). A single dimension of semantic relatedness may entangle various relations and dilute semantic effects to be observed.

**Figure 1:**
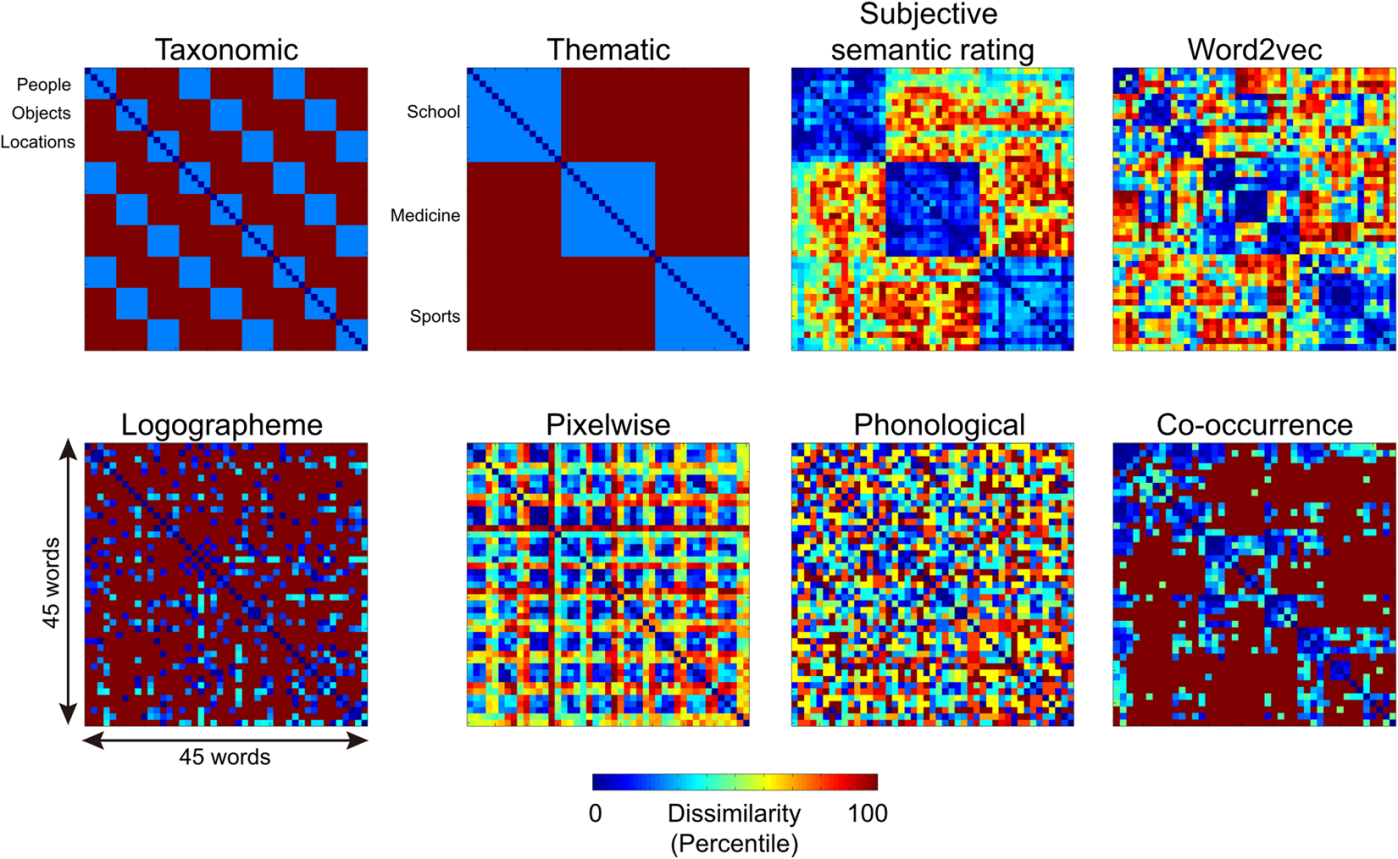
Theoretical/behavioral representational dissimilarity matrices. The binary taxonomic and thematic RDMs illustrated the membership of each word in three taxonomic categories (people, objects, and locations) and three thematic categories (school, medicine, and sports). The subjective semantic rating RDM was based on explicit ratings of semantic distance. The word2vec RDM was calculated as the cosine distance of vector representations of words learned in a skip-gram model. The logographeme, pixelwise and phonological RDMs were constructed by one minus the proportion of shared logographemes, overlapping pixels for visual words in a pictorial format and shared sub-syllabic units and tones, respectively, for a given word pair. The co-occurrence RDM was constructed based on the summed counts of co-occurrence within a window of five words for a given word pair in a language corpus.

In this study, to examine whether and how the VWFA activity is influenced by semantic processing in reading comprehension, we tested multiple types of semantic relations in the VWFA in tasks requiring explicit semantic access. Specifically, we asked participants to perform taxonomic or thematic categorization tasks on the 45 words that fell into nine conditions arising from the combinations of three taxonomic categories (people, manmade objects, and locations) and three thematic categories (school, medicine, and sports), while obtaining the blood-oxygen-level-dependent (BOLD) fMRI responses to each word. We constructed semantic representational dissimilarity matrices (RDMs) based on four types of semantic relations: taxonomic, thematic, subjective semantic rating and word2vec^25^ (a computational linguistic measure based on word co-occurrence patterns in a large language corpus) (Figure 1, top panel). Taxonomic and thematic RDMs targeted at specific dimensions of the semantic space by indicating whether the two words belonged to the same taxonomic or thematic category. The subjectively rated semantic distance and word2vec RDMs measured composite semantic relationships, which may integrate multiple dimensions of relatedness. The representational similarity analysis (RSA) approach^26^ was adopted to evaluate the representational content of the VWFA by correlating the semantic RDMs with the neural RDMs derived from the word-word correlation distance embedded in neural patterns in each task. Orthographic, low-level visual and phonological RDMs were constructed and controlled for in further analyses to rule out the possibility that any semantic effects may be driven by these non-semantic information types in this region.

## Results

### Relationships among the theoretical/behavioral RDMs

Figure 1 illustrates eight theoretical/behavioral RDMs constructed by the pairwise relations of the 45 words, including four semantic RDMs (top panel, see Introduction) and four non-semantic RDMs (bottom panel, see Methods). The logographeme, pixelwise and phonological RDMs characterized word-word dissimilarity in orthographical, low-level visual and phonological information, respectively. The co-occurrence RDM measured how likely the two words would appear together in a 5-word window in texts, which might reflect the co-occurrence statistics in the visual field.

The Spearman correlation coefficients among these RDMs are shown in Table 1. Among the four semantic RDMs, the taxonomic and thematic RDMs were not correlated due to careful selection of stimuli. The two composite semantic RDMs (subjective rating and word2vec) were significantly correlated with each other (*r* = 0.441, *P* < 10^−10^) and differed in how they related to the taxonomic and thematic RDMs. The subjectively rated semantic distance was strongly correlated with the thematic RDM (*r* = 0.798, *P* < 10^−10^), not with the taxonomic RDM (*r* = 0.017, *P* = 0.584), whereas the word2vec distance showed significant correlations with both taxonomic and thematic RDMs (*r*s > 0.422, *P*s < 10^−10^). For the relations between semantic and non-semantic RDMs, all the semantic RDMs were significantly correlated with the co-occurrence RDM (*r*s > 0.189, *P*s < 10^−8^), implying that visual co-occurrence may be a confounding variable in any observed semantic effects. The word2vec distance was also significantly correlated with the logographeme and pixelwise RDMs (*P*s < 10^−4^), which is consistent with the notion that this algorithm captures multiple dimensions of similarity^25^. The significant correlation between taxonomic and logographeme RDMs (*r* = 0.107, *P* = 0.0007) is likely to be due to the fact that the majority of Chinese characters are so-called composite characters, containing a semantic radical and a phonological radical. Characters belonging to the same taxonomic category sometimes share the same semantic radical. For instance, many animal words in Chinese share the semantic logographeme “”, e.g. “”(cat), “”(dog), “” (wolf), “” (fox)). The correlations between pixelwise and co-occurrence (*r* = 0.166, *P* < 10^−6^), pixelwise and phonological (*r* = −0.105, *P* = 0.0009) RDMs are less straightforward to interpret and might be epiphenomenal in Chinese written language given the prevalence of orthographic neighbors/homophones of Chinese characters.

**Table 1:** Spearman correlation coefficients among theoretical/behavioral representational dissimilarity matrices.

		Semantic RDMs				Non-semantic RDMs			
		Taxonomic	Thematic	Subjective rating	word2vec	Logographeme	Pixelwise	Co-occurrence	Phonological
Semantic RDMs	Taxonomic	1	−0.048	0.017	0.422	0.107	−0.002	0.189	0.056
	Thematic		1	0.798	0.492	0.060	−0.024	0.424	0.022
	Subjective rating			1	0.441	0.049	0.049	0.572	0.034
	word2vec				1	0.139	−0.206	0.306	0.083
Non-semantic RDMs	Logographeme					1	0.035	0.043	0.095
	Pixelwise						1	0.166	−0.105
	Co-occurrence							1	0.057
	Phonological								1

Note: Number in bold indicates Bonferroni-corrected *P* < 0.05 (two-tailed).

### Behavioral results in the fMRI experiment

In the scanner, participants were presented with the 45 words that fell into nine conditions arising from the combinations of three taxonomic categories (people, manmade objects, and locations) and three thematic categories (school, medicine, and sports), with five words per condition (see Supplementary Table S1). In different runs, they were asked to categorize each word either by taxonomic or thematic memberships. They performed the two tasks with equally high accuracy (taxonomic task, mean = 96%, standard deviation (SD) = 3%; thematic task, mean = 96%, SD = 3%; task difference, paired *t*_18_ = 0.28, *P* = 0.78) and with comparable reaction times (taxonomic task, mean = 1513.50 ms, SD = 415.33 ms; thematic task, mean = 1497.03 ms, SD = 437.34 ms; task difference, paired *t*_18_ = 0.46, *P* = 0.65).

### Orthographic representation in the VWFA

We defined the VWFA in both anatomical and functional ways. To verify orthographic representation in the anatomically defined VWFA and to functionally localize voxels sensitive to orthography, we examined the correspondence between the logographeme RDM and the neural RDMs based on the overall functional data (i.e. the collapsed dataset of the taxonomic and thematic tasks, see Methods).

#### Anatomically defined VWFA

**Figure 2:**
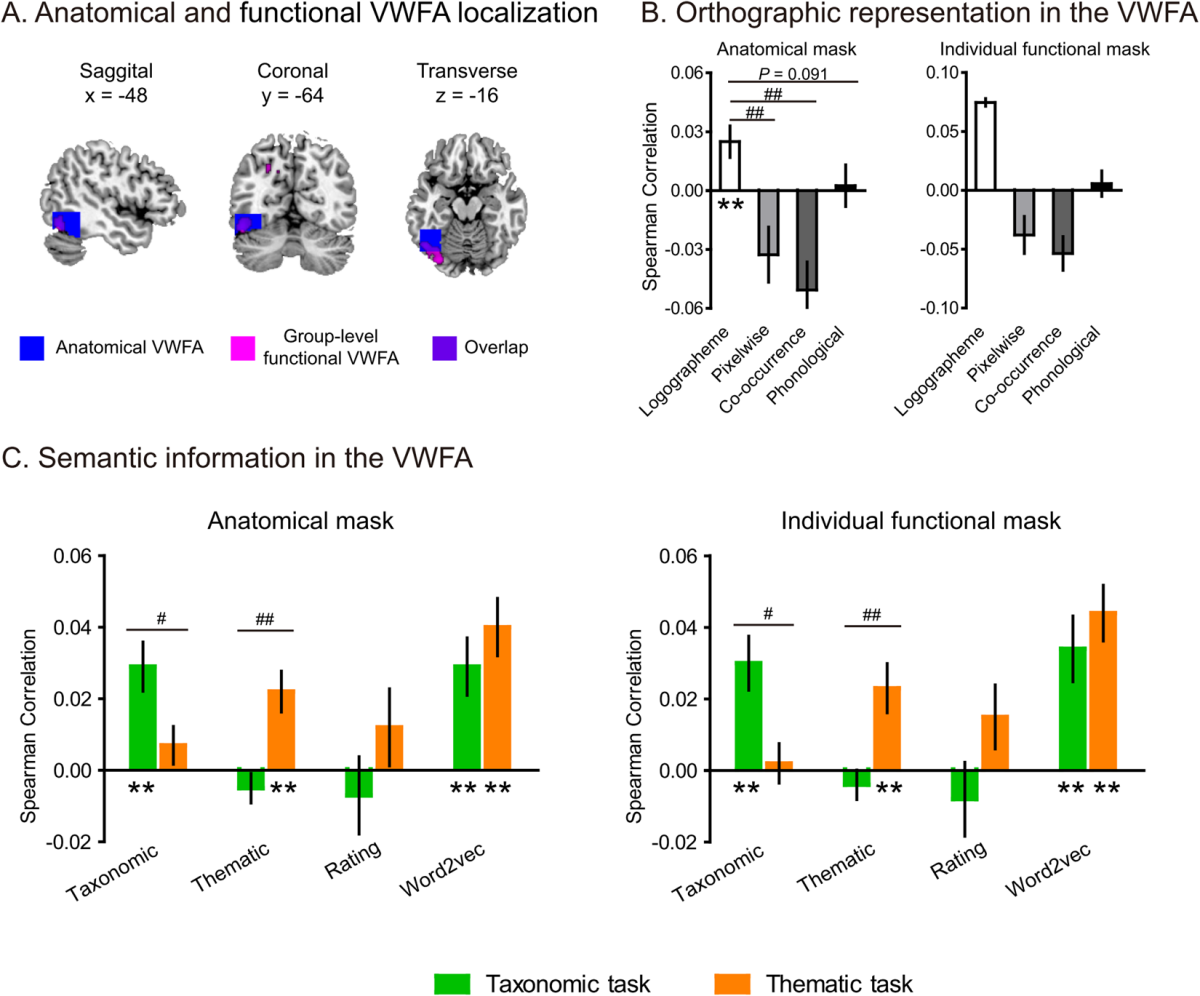
Orthographic and semantic information in the VWFA. (**A**) Anatomical and group-level functional VWFA localization. The anatomical mask was a box covering the left posterior occipitotemporal sulcus^27^; the group-level functional VWFA was localized using a whole-brain searchlight RSA with the logographeme RDM (cluster-level FWE corrected *P* < 0.05, peak MNI coordinates *xyz*: −48, −64, −16). The two masks showed certain overlap. (**B**) Orthographic representation in the VWFA. (**C**) Specific and composite semantic information in the VWFA. The bars report the averaged Fisher-*z*-transformed Spearman correlation between individual subject’s neural RDMs in each VWFA mask and the theoretical/behavioral RDMs. Error bar: ±standard error. **P < 0.01, one-sample *t* tests against zero (one-tailed); ^**#**^*P* < 0.05, ^**##**^*P* < 0.01, paired *t* tests (two-tailed).

The anatomical mask was defined as a box covering the left posterior occipitotemporal sulcus with cerebellum voxels excluded (Figure 2A)^27^. One-sample *t* tests (one-tailed) revealed significantly positive correlation for logographeme information in this region of interest (group-averaged Fisher-*z*-transformed Spearman *r* (mean *r*) = 0.025; *t*_18_ = 3.112, *P* = 0.003), but not for pixelwise, co-occurrence, or phonological information (*P*s > 0.41; Figure 2B, left panel). Comparing correlation coefficients of logographeme information with each of these control variables revealed significant differences between logographeme and pixelwise information (paired *t*_18_ = 3.380, *P* = 0.003), between logographeme and co-occurrence information (paired *t*_18_ = 4.024, *P* < 0.001). The difference between logographeme and phonological information showed a nonsignificant trend toward significance (paired *t*_18_ = 1.788, *P* = 0.091).

#### Functionally defined VWFA

For the functional VWFA localization, a whole-brain searchlight RSA with the logographeme RDM (cluster-level FWE corrected *P* < 0.05, voxelwise *Z* > 3.09) revealed one single cluster in the left posterior occipitotemporal cortex (peak MNI coordinates *xyz* = −46, −64, −18; for the other two significant regions see Table 2), which partially overlapped with the anatomically defined VWFA (Figure 2A). We then identified functional VWFA in individual subjects using the same way (see Methods) and examined its encoding of pixelwise, co-occurrence and phonological information (Figure 2B, right panel). One-sample *t* tests (one-tailed) revealed that none of the three types of information was significantly associated with the neural activity patterns of the functional VWFA (*P*s > 0.30).

**Table 2:** Brain regions whose activity patterns encoded logographeme information of Chinese words in a whole-brain searchlight analysis (cluster-level FWE corrected *P* < 0.05 with voxelwise *Z* > *3.09*).

Anatomical Label	Cluster Size (Voxels)	P_FWE-Corr_	Peak Voxel	MNI Coordinates		
		(Cluster Level)	(Pseudo *t* Value)	x	y	Z
L Fusiform	313	0.037	4.221	−48	−64	−16
L Superior Parietal	280	0.043	4.538	−12	−72	42
R Precentral	357	0.030	4.217	32	−24	54

#### Task modulation effects

We investigated whether semantic demands could modulate the orthographic representation in the VWFA by comparing the correlations of the logographeme and neural RDMs between the two tasks. We first looked at logographeme representation in the VWFA in each task and found that while the neural activity patterns of the functional VWFA significantly associated with orthographic information in both tasks (taxonomic task: mean *r* = 0.030, *t*_18_ = 6.014, *P* < 0.001; thematic task: mean *r* = 0.030, *t*_18_ = 5.130, *P* < 0.001), the anatomically defined VWFA showed weaker representations (taxonomic task: mean *r* = 0.008, *t*_18_ = 1.463, *P* = 0.080; thematic task: mean *r* = 0.009, *t*_18_ = 1.028, *P* = 0.159), possibly due to the imprecise localization of orthography-sensitive voxels in individuals. Nevertheless, in both VWFA masks, paired *t* tests comparing logographeme information between the two tasks revealed no significant differences (*P*s > 0.922), indicating task-independent orthographic representation in this region.

### Semantic information in the VWFA

We then examined how various types of semantic information were encoded in the VWFA in each task and how they were modulated by task demands (Figure 2C).

#### Taxonomic information

In the taxonomic task, the taxonomic RDM showed significantly positive correlation with the neural RDM in the VWFA regardless of the mask definition (anatomical mask: mean *r* = 0.029, *t*_18_ = 4.166, *P* < 0.001; functional mask: mean *r* = 0.030, *t*_18_ = 3.890, *P* = 0.001). In the thematic task, the taxonomic information was not associated with the activity patterns of the VWFA in either mask (*P*s > 0.115). Significant task differences were found in both the anatomical (paired *t*_18_ = 2.302, *P* = 0.033) and functional (paired *t*_18_ = 2.790, *P* = 0.012) VWFA masks.

#### Thematic information

In the taxonomic task, the thematic RDM was not correlated with the neural RDM of the VWFA in either mask (*P*s > 0.338). In the thematic task, the thematic information showed significantly positive correlation with the neural RDM in the VWFA (anatomical mask: mean *r* = 0.022, *t*_18_ = 3.773, *P* < 0.001; functional mask: mean *r* = 0.023, *t*_18_ = 3.242, *P* = 0.005). Significant task differences were found in both the anatomical (paired *t*_18_ = 3.261, *P* = 0.004) and functional (paired *t*_18_ = 3.051, *P* = 0.007) VWFA masks.

#### Subjective semantic rating

In the taxonomic task, the subjectively rated semantic RDM was not associated with the neural RDM of either the anatomical or functional VWFA masks (*P*s > 0.737). In the thematic task, the presence of this information in the VWFA approached significance (anatomical mask: mean *r* = 0.012, *t*_18_ = 1.468, *P* = 0.080; functional mask: mean *r* = 0.015, *t*_18_ = 1.645, *P* = 0.059). Direct comparison between the two tasks did not reveal significant differences (*P*s > 0.154).

#### Word2vec

The word2vec RDM was significantly associated with the neural RDM of the VWFA in both the taxonomic task (anatomical mask: mean *r* = 0.029, *t*_18_ = 3.541, *P* = 0.001; functional mask: mean *r* = 0.034, *t*_18_ = 3.701, *P* < 0.001) and the thematic task (anatomical mask: mean *r* = 0.040, *t*_18_ = 4.731, *P* < 0.001; functional mask: mean *r* = 0.044, *t*_18_ = 5.591, *P* < 0.001). No significant task differences were observed (*P*s > 0.311).

### Semantic information encoded in the VWFA: Controlling for non-semantic confounding variables

To test whether semantic information can explain the variance of the neural activity patterns in the VWFA over and above non-semantic factors, we computed partial correlations between the neural and semantic RDMs, controlling for the logographeme, pixelwise, co-occurrence and phonological RDMs. As shown in Table 3, task-relevant semantic information and word2vec distance remained significant in these partial correlation analyses. The subjectively rated semantic distance was (marginally) significant in the thematic task when the co-occurrence RDM was included as a nuisance variable. Linear regression analyses were then carried out to examine the unique contribution of orthography and semantic information to the neural RDM of the functionally defined VWFA (the anatomically defined VWFA was not analyzed here due to the insignificant orthographic representation in each task). Taking the group-averaged neural RDM as the dependent variable and the logographeme, taxonomic and thematic RDMs as the independent variables, we found that in the taxonomic task the logographeme (β = 0.126, *P* = 0.003) and taxonomic (β = 0.105, *P* = 0.008) information were significant predictors of the neural RDM, whereas in the thematic task the logographeme (β = 0.135, *P* = 0.001) and thematic (β = 0.086, *P* = 0.028) information were significant predictors. These results suggest the joint presence of orthographic and task-relevant semantic information in the VWFA in semantic tasks.

**Table 3:** Group-averaged Fisher-*z*-transformed Spearman correlation coefficients for semantic information in the VWFA, controlling for non-semantic confounding variables.

	Anatomical VWFA				Individual functional VWFA			
	Taxonomic	Thematic	Rating	word2vec	Taxonomic	Thematic	Rating	word2vec
*Controlling for the logographeme RDM*								
Taxonomic task	**0.028**	−0.006	−0.007	**0.028**	**0.027**	−0.006	−0.009	**0.030**
Thematic task	0.006	**0.022**	0.012	**0.039**	−0.001	0.021^#^	0.013	**0.040**
*Controlling for the logographeme and pixelwise RDMs*								
Taxonomic task	**0.028**	−0.006	−0.008	**0.030**	**0.027**	−0.006	−0.009	**0.031**
Thematic task	0.005	**0.021**	0.013	**0.034**	−0.002	0.020^#^	0.015	**0.033**
*Controlling for the logographeme, pixelwise and co-occurrence RDMs*								
Taxonomic task	**0.040**	0.011	0.015	**0.047**	**0.037**	0.012	0.015	**0.046**
Thematic task	0.012	**0.031**	0.030^#^	**0.046**	0.005	**0.029**	**0.031**	**0.046**
*Controlling for the logographeme, pixelwise, co-occurrence and phonological RDMs*								
Taxonomic task	**0.040**	0.011	0.015	**0.047**	**0.037**	0.012	0.015	**0.046**
Thematic task	0.012	**0.031**	0.030^#^	**0.046**	0.005	**0.029**	**0.031**	**0.046**

Note: Number in bold indicates Bonferroni-corrected *P* < 0.05 (one-sample *t* test, one-*t*ailed). ^**#**^Indicates Bonferroni-corrected *P* < 0.1 (one-sample *t* test, one-tailed).

## Discussion

The aim of this study was to investigate whether the VWFA activity encodes semantic information in explicit semantic tasks. Using RSA, we computed the correlations between RDMs derived from neural activity patterns in the VWFA with various types of semantic RDMs in two semantic categorization tasks–taxonomic and thematic categorization. We found that the VWFA activity patterns were modulated by the semantic tasks, with words’ neural RDMs showing significant association with semantic dimensions that were relevant for the specific task being performed. That is, words that are taxonomically related (e.g. teacher-doctor) had more similar VWFA activity patterns under the taxonomic categorization task (people, objects, or locations) and those that are thematically related (e.g. teacher-classroom) had more similar VWFA activity patterns under the thematic categorization task (school, medicine, or sports). The composite semantic similarity measure derived from the advanced natural language processing algorithm (i.e., word2vec) together with big-data language corpora showed significant effects in both semantic categorization tasks, so did the orthographic similarity (the logographeme RDM). These findings provide positive evidence that both orthographic and semantic information was encoded in the VWFA during semantic processing and that the semantic effect dimensions change with task goals.

We first verified that the activity pattern in the left posterior occipitotemporal cortex is sensitive to the orthographic similarity of Chinese words. By constructing an orthographic RDM based on the overlap of logographemes–the basic functional unit in Chinese characters^28^–between words, we found that the logographeme RDM showed significantly positive correlations with the neural RDM in the pre-defined anatomical mask and localized a cluster in the same region in the whole-brain searchlight analysis. Together with previous findings of orthographic representations in this region using RSA^6–8^, this line of evidence echoes neuroimaging studies with conventional univariate approaches^3,5,19^ and lesion studies^11^ in supporting the central role of the VWFA in the orthographic representation. Orthographic computation appears to be an inherent property of the VWFA, because of either its sensitivity to specialized orthographic inputs^17,29^ and/or synthesis of bottom-up inputs and top-down predictions^18^ and is thus robust regardless of tasks.

The effects of semantics in the VWFA are more complex. We did find positive semantic effects, but the effects varied by the type of semantic dimensions. For specific dimensions including thematic and taxonomic relations, the organization was tuned according to that particular dimension being judged. For subjectively rated semantic similarity measure, we did not see any significant effects, except for a trend in the thematic categorization task. For the semantic similarity derived from large-scale text using statistical learning models (word2vec), the effects were present in both semantic tasks. Worth noting is that semantic effects in the VWFA cannot be explained by the orthographic, low-level visual, first-order co-occurrence and phonological effects. Among these variables that were excluded from explaining the semantic effects, the first-order co-occurrence RDM is of particular interest. This model can be considered as an extended version of orthographic representation by characterizing how likely two word forms would co-occur in a local visual context (five words) during natural reading. Semantically related words (in both specific and composite semantic RDMs) tend to visually co-occur (Table 1), raising the possibility that semantic effects could be ascribed to visual co-occurrence in reading. Nevertheless, RSA results showed that words with greater first-order co-occurrences did not evoke more similar activation patterns in the VWFA (Figure 2B) and, more importantly, semantic effects remained significant when the first-order co-occurrence measure was controlled for. That is, the semantic effects in the VWFA we observed are not explained by these non-semantic properties we tested.

Why are there task-sensitive dimension-specific semantic effects in the VWFA and why are the word2vec effects present in both tasks? One possibility is that the VWFA contains neuronal populations sensitive to both taxonomic and thematic organizations. Attention boosts task-relevant information and/or tune down task-irrelevant information so that only task-relevant information is observed in the VWFA activity^30^. Such semantic information, even if present, seems to be subtle or redundantly coded in other regions, as lesion/disruption to the VWFA had minimal influence on object recognition and language comprehension abilities^12^. Another scenario consistent with the broader empirical findings is that the VWFA itself does not store semantic information, but inherits activation patterns in the higher-order semantic regions via top-down feedback. In semantic judgment tasks, when a reader sees the word “teacher”, the visual input activates its orthographic representation (likely to be in the VWFA) and then the corresponding word meaning representation (stored somewhere else in the semantic neural system). The semantic representations that are related to the target meaning (e.g. “doctor” or “classroom”) along various dimensions are also activated through spreading of activation due to overlapping features or associations. The types of neighboring meanings receiving more activation are dependent on the task at hand – when the judgment is about taxonomic classes, the taxonomic neighbors are more strongly activated; when the judgment is about thematic relations, the thematic neighbors are more strongly activated. Such activated semantic neighboring representations in turn feeds back to their own orthographic representations in the VWFA, resulting in more similar VWFA activity patterns for items sharing that semantic dimension. Such feedback does not seem simply epiphenomenal, but may contribute to orthographic identification^31^ and overall task performance. Given the distributed neural basis of semantic memory^23,32^, future studies are warranted to uncover the specific mechanisms of modulation between semantic regions and the VWFA using approaches that are optimized to study task-specific functional connectivity patterns.

Our study highlights the importance of taking the multidimensional and dynamic nature of semantic information into account when investigating the neural correlates of semantic processing. Previous studies that used subjective semantic relatedness have reported null results for semantic effects in the VWFA^3,4^. Our rating results showed that the subjectively rated semantic RDM tended to be more similar to the thematic RDM than the taxonomic RDM, indicating that in our free rating context, the group-level subjectively perceived semantic distance is biased towards thematic relations. This is consistent with a similar preference for thematic thinking in the matching or free association tasks and accords with the impact of thematic relations on word similarity judgment^33^. Thus, the semantic effects based on such measures may not be detectable in semantic tasks that do not rely on such dimension, e.g., detecting certain taxonomic categories^3^. In comparison, the word2vec distance was found to correlate with both the taxonomic and thematic RDMs, indicating that this composite semantic space is a multidimensional one that captures both taxonomic and thematic relations, thus explaining the results that the word2vec RDM correlated with the VWFA neural activity in both tasks. This is consistent with the marginally significant effect of the LSA distance–another composite measure containing both types of relations^34,35^. The significant effects of word2vec in our study may be because word2vec captures richer semantic information than LSA^25^.

The significant semantic effects observed here, in comparison to previous studies, are also likely to be driven by the explicit semantic tasks we used. For tasks where (deep) semantic processing was not necessary such as lexical decision, semantic effects tended not to be consistent in the VWFA^3,4,7^. To our knowledge, there was only one study reporting both orthographic and semantic effects in the primed lexical decision task in the posterior fusiform gyrus^19^. In that study, the target word was presented 1300 ms, a period long enough for participants to explicitly associate it with the visible prime (presented for 150 ms). This is in contrast with other priming studies using very short stimulus representation time that emphasizes bottom-up input properties (e.g. 300 ms^3,4^). Therefore, it seems that explicit and detailed semantic processing, as well as the consistency between semantic contents and task demands, would be required for robust semantic effects in the VWFA.

To conclude, by including multiple types of semantic distance measures and different task demands, we demonstrate that in explicit semantic tasks the activity patterns of the VWFA also contain task-relevant semantic information of written words in addition to orthographic information. Future studies are warranted to examine how semantic processing in the VWFA interacts with orthographic representations to support fluent reading.

## Methods

### Subjects

Twenty young healthy adults recruited from Peking University participated in this study (10 males; aged 18–27 years). They were all right handed, native Chinese speakers, with normal or corrected-to-normal vision. The study was approved by the Human Subject Review Committee at Peking University. All the experiments were performed in accordance with relevant guidelines and regulations. Informed consent was obtained from all participants. One participant was excluded from data analysis due to recoding errors of button press.

### Stimuli and fMRI procedure

The stimuli set contained 45 Chinese words (see Supplementary Table S1) that belonged to nine conditions arising from the combinations of 3 taxonomic categories (people, manmade objects, and locations) and 3 thematic categories (school, medicine, and sports), with five exemplar words per condition. Three out of five words were bisyllabic (two characters) and the other two trisyllabic (three characters). Before scanning, participants were shown pictures of the intended meaning of each word to reduce word meaning ambiguity when words are presented alone.

The condition-rich rapid event-related design was adopted for the fMRI scan^26^, with each word as an experimental condition. Lasting 260 s, each run started and ended with a 10 s blank screen and included 45 word trials, with each word presented exactly once. Each word trial started with a fixation cross on the center of a gray background for 500 ms, followed by the word (Song bold font, 36 point in font size) for 500 ms and a blank screen with varying lengths between 3 and 13 s. The duration of the blank screen as well as the stimulus sequence (organized as nine conditions) were determined using the optseq 2 optimization algorithm^36^. Five words within each condition were randomly presented and run orders were further randomized across participants. There were 10 runs in total.

Two semantic categorization tasks were adopted. In half of the runs, a taxonomic judgment task was performed, in which participants were asked to categorize each word into three taxonomic categories (people, objects, and locations) by pressing three buttons with their right middle finger, right index finger and left index finger, respectively. In the other half of the runs, participants performed a thematic judgment task in which they categorized words into three thematic categories (school, medicine, and sports) using the same fingers and buttons in the taxonomic task. The run order of taxonomic and thematic tasks was randomized across participants.

### fMRI acquisition and preprocessing

The fMRI results were reanalyses of data that were collected for another study investigating the neural basis of semantic relations. The acquisition and preprocessing procedures are as follows. Whole-brain imaging was performed on a 3 T Siemens MRI Scanner (MAGNETOM Prisma) at the Center for MRI Research, Peking University. Functional images were acquired using the multi-band echo-planar sequence [repetition time (TR) = 2000 ms, echo time (TE) = 30 ms, flip angle (FA) = 90°, matrix size = 112 × 112, 64 axial slices, voxel size = 2 × 2 × 2.2 mm, multi-band factor = 2]. High-resolution three-dimensional T1-weighted images were acquired using the magnetization-prepared rapid gradient-echo sequence (TR = 2530 ms, TE = 2.98 ms, inversion time = 1100 ms, FA = 7°, matrix size = 448 × 512, 192 sagittal slices, voxel size = 0.5 × 0.5 × 1 mm).

The images were preprocessed using SPM12 (Wellcome Trust Center for Neuroimaging, http://www.fil.ion.ucl.ac.uk/spm/software/spm12/). For each participant data, after discarding the first five volumes of each run, functional images were corrected for slice timing and head motion. The resulting un-smoothed and un-normalized images were entered into the general linear model (GLM) for further analysis. The structure image was co-registered to the mean functional images and segmented into different tissues. The deformation fields for spatial normalization of native space to the Montreal Neurological Institute (MNI) space and reverse normalization were also obtained in this step.

### fMRI data analysis

The whole-brain activation maps for each word in individual subjects were obtained via GLM in the first-level analysis. Two GLMs were built, differing on whether to include task-specific regressors for each word. The first GLM included 45 regressors for each run, one for each word and and the second GLM included 90 regressors, two for each word with one for the taxonomic task and the other for the thematic task. Trial-level differences in reaction time (RT) were controlled for by convolving each trial with a boxcar equal to the length of its reaction time^37^. Six head motion parameters and a global mean predictor for each run were also included in GLMs. A high-pass filter cut-off was set at 128 s. The subsequent word versus baseline contrast produced a whole-brain *t* map for each word and for each word under each task, which was used for the following activation pattern analyses.

### Representational similarity analysis

RSA is a widely used approach to characterize the correspondence between brain activity patterns and theoretical/behavioral measurement^38^. This method consists of constructing representational dissimilarity matrices (RDMs) for both measures and calculating the correlation between them. An RDM is a symmetric *n* × *n* matrix, where *n* is the number of experimental conditions (*n* = 45 in this study) and the off-diagonal values indicate the dissimilarity (or distance) for each pair of conditions in a certain aspect.

#### Theoretical/behavioral RDMs

Four semantic RDMs were constructed to investigate the potential semantic information embedded in the activity patterns of the VWFA. The **taxonomic RDM** was a binary RDM, assigning 0 to word pairs that belong to the same taxonomic category (e.g. teacher-doctor, chalk-bandage, classroom-hospital) and 1 to the remaining cells. The **thematic RDM** was also a binary RDM, assigning 0 to word pairs that belong to the same thematic category (e.g. teacher-student, teacher-chalk, teacher-classroom) and 1 to the remaining cells. The **subjectively rated semantic RDM** was based on pairwise ratings of semantic distance. Eighteen healthy college students (nine females, mean age = 23.5 years, range = 18–27 years) were recruited to rate how close two words were in meaning using a 7-point Likert scale (7 for the closest). Ratings for a total of 990 word pairs (pairwise combination of the 45 words) were collected and the RDM was computed as seven minus the averaged rating scores of 18 participants for each word pair, which resulted in a symmetric 45 × 45 matrix. The **word2vec RDM** was based on continuous vector representations of words generated by the skip-gram architecture^25^. For the Baidu encyclopedia corpus containing approximately one billion word tokens, a vocabulary of the most frequent 249,222 words was first obtained through the Stanford parser. The word2vec tool was then used to train vector representations of words (https://code.google.com/p/word2vec/) with the following parameters: window size = 5, sub-sampling rate = 10^−4^, negative sample number = 5, learning rate = 0.025, dimension number = 300. The word distance was measured as one minus the cosine angle between feature vectors of each word.

To validate that the VWFA activity patterns are sensitive to orthographic information, we constructed a **logographeme RDM** to characterize orthographic dissimilarity between words. The logographeme has been proposed to be the basic unit of Chinese characters^28,39^. The logographeme RDM was measured by one minus the proportion of shared logographemes between two words regardless of position. For instance, the word “” (campus) is composed of seven logographemes (“); the word “”(tampon) is composed of five logographemes (“”). They shared one logographeme (“”) and therefore the dissimilarity is 1-(1/12) = 0.917. Three control RDMs were constructed. The **visual pixelwise RDM** measured the pixelwise overlap of the binary silhouette images of word pairs^7,38^. The **co-occurrence RDM** measured how likely the two words would appear together in a 5-word window in texts and was based on summed counts of co-occurrence frequency for each word pair within five words in the Chinese Web 5-gram Corpus (https://catalog.ldc.upenn.edu/LDC2010T06), which contains about 883 billion word tokens extracted from Chinese Web pages. The co-occurrence counts were log-transformed using ln (f + 40), where f is the raw summed counts and 40 is the lowest n-gram counts kept in the database, and then reversed to construct the co-occurrence RDM. The **phonological RDM** was calculated as one minus the proportion of shared sub-syllabic (initials or finals) units and tones regardless of position.

#### VWFA localization

The VWFA was defined in both anatomical and functional ways. An *a priori* anatomical mask covering the posterior occipitotemporal sulcus was adopted^27^. This mask ranged from −54 < x < −30, −70 < y < −45 and −30 < z < −4 in the MNI space and voxels in the cerebellum according to the automated anatomical labeling template were excluded^40^. This mask was reverse-normalized into each subject’s native space for further analysis.

To localize functional VWFA, a whole-brain searchlight RSA^41^ was first performed to identify brain regions sensitive to logographeme information. For each voxel in native space, we built a spherical region of interest (ROI, radius 6 mm) centering on the voxel, extracted *t* values in this ROI to each of the 45 words and calculated one minus Spearman rank correlations of all word pairs within this ROI to construct a neural RDM. The relationship between the neural RDM and the logographeme RDM was then assessed using partial Spearman correlation with the visual pixelwise RDM being controlled for (to ensure that orthographic representation was not contaminated by low-level visual similarity), which produced a correlation coefficient for this voxel. Moving the searchlight center throughout the cortex, we obtained a whole-brain *r*-map in the native space. Note that the searchlight analysis was restricted to the voxels with a probability higher than 1/3 in the native gray matter image generated from the segmentation step. For a group-level random-effects analysis, the *r* maps in the native space were Fisher-*z*-transformed, normalized to the MNI space using the forward deformation field and spatially smoothed using a 6 mm full-width at half maximum Gaussian kernel. The permutation-based statistical non-parametric mapping (SnPM; http://go.warwick.ac.uk/tenichols/snpm) was used (no variance smoothing, 10,000 permutations) to test for significance of positive correlations between the neural and logographeme RDMs across participants. Clusters surviving the cluster-level FWE correction at *P* < 0.05 with a voxelwise threshold of *Z* > 3.09 were reported. A single cluster was found in the left posterior occipitotemporal cortex, partially overlapping with the anatomical mask of the VWFA and was defined as the group-level functional VWFA (see Figure 2A and results).

For each subject, we then identified the voxels in the anatomical mask of the VWFA whose neural RDMs showed a significantly positive correlation with the logographeme RDM in the above-mentioned searchlight analysis (one-tailed *P* < 0.05, uncorrected; mean number of voxels across participants, 108, range: 10–302 voxels). These voxels together with their adjacent voxels within a 6-mm-radius sphere were considered as individual subjects’ functional VWFA (mean number of voxels: 1362, range: 349–2590 voxels).

#### RSA procedures for the VWFA

For both anatomical and functional VWFA masks, we calculated the neural RDMs as one minus Spearman’s rank correlation between each pair of words. To validate that the activation patterns of the VWFA showed some specificity of orthographic information, we first calculated the Spearman correlation between the neural RDM and the logographeme, visual pixelwise, co-occurrence and phonological RDMs for each ROI (Note that the logographeme effect of the functional ROI was shown for illustration purposes). We then investigated the semantic information in the VWFA in detail. Specifically, the neural RDMs for each task were compared with four semantic RDMs using the Spearman rank correlation. Partial correlations were also performed to control for logographeme, visual pixelwise, co-occurrence and phonological RDMs. The resulting correlation coefficients were Fisher-*z*-transformed and statistically inferred across participants. One-sample *t* tests were used to test whether the correlation was significantly greater than zero. Paired *t* tests were used to compare different information types and the same information type in different tasks.

### Data availability

The datasets generated and/or analyzed during the current study are available from the corresponding authors on reasonable request.

## Electronic supplementary material

Supplementary information
