# Supplementary material for: Representational similarity analysis reveals task-dependent semantic influence of the visual word form area
Source: Sci Rep. 2018 Feb 14;8:3047. doi: 10.1038/s41598-018-21062-0 (PMC5813029; doi:10.1038/s41598-018-21062-0)
Supplement: Supplementary file 1 — Supplementary information [file 41598_2018_21062_MOESM1_ESM.docx]

**Title:** Representational similarity analysis reveals task-dependent semantic influence of the visual word form area

**Authors:** Xiaosha Wang^1,2,3#^, Yangwen Xu^2,3#^, Yuwei Wang^4,5^, Yi Zeng^4,5^, Jiacai Zhang^1*^, Zhenhua Ling^6*^, Yanchao Bi^2,3^

**Affiliations:** 1. College of Information Science and Technology, Beijing Normal University, Beijing, 100875, China;

2. National Key Laboratory of Cognitive Neuroscience and Learning & IDG/McGovern Institute for Brain Research, Beijing Normal University, Beijing, 100875, China;

3. Beijing Key Laboratory of Brain Imaging and Connectomics, Beijing Normal University, Beijing, 100875 China;

4. Research Center for Brain-inspired Intelligence & National Laboratory of Pattern Recognition, Institute of Automation, Chinese Academy of Sciences, Beijing, 100190, China;

5. Center for Excellence in Brain Science and Intelligence Technology, Chinese Academy of Sciences, Shanghai, 200031 China;

6. National Engineering Laboratory for Speech and Language Information Processing, University of Science and Technology of China, Hefei 230027, China;

**Correspondence**: Zhenhua Ling, National Engineering Laboratory for Speech and Language Information Processing, University of Science and Technology of China, Hefei 230027, China; Tel/Fax: 86-551-63603140; E-mail: zhling@ustc.edu.cn. Jiacai Zhang, College of Information Science and Technology, Beijing Normal University, Beijing, 100875, China; Tel/Fax: 86-10-58807856/58800056; E-mail: jiacai.zhang@bnu.edu.cn.

#: these authors contributed equally to the work.

**Supplementary Table S1**

*Forty-five words used in this study (Chinese words and English translations, organized by category).*

|  | | Thematic categories | | |
| --- | --- | --- | --- | --- |
|  |  | School | Medicine | Sports |
| Taxonomic categories | People | 老师 (teacher), 学生 (student), 教授 (professor), 班主任 (class advisor), 课代表 (subject representative) | 医生 (doctor), 病人 (patient), 护士 (nurse), 麻醉师 (anesthetist), 助产士 (midwife) | 裁判 (referee), 教练 (coach), 观众 (audience), 运动员 (athlete), 拉拉队 (cheerleader) |
|  | Objects | 直尺 (ruler), 书包 (schoolbag), 粉笔 (chalk), 橡皮擦 (eraser), 作业本 (exercise book) | 针筒 (injector), 棉球 (tampon), 绷带 (bandage), 止血钳 (haemostatic forceps), 听诊器 (stethoscope) | 铁饼 (discus), 铅球 (shot), 标枪 (javelin), 接力棒 (relay baton), 发令枪 (starting gun) |
|  | Locations | 校园 (campus), 教室 (classroom), 讲台 (rostrum), 自习室 (study lounge), 图书馆 (library) | 医院 (hospital), 病房 (sickroom), 药房 (pharmacy), 门诊楼 (outpatient building), 手术室 (operating theater) | 跑道 (track), 看台 (bleacher), 沙坑 (jumping pit), 体育场 (stadium), 领奖台 (award platform) |
